# Toxicity of unsaturated fatty acids to the biohydrogenating ruminal bacterium, *Butyrivibrio fibrisolvens*

**DOI:** 10.1186/1471-2180-10-52

**Published:** 2010-02-18

**Authors:** Margarida RG Maia, Lal C Chaudhary, Charles S Bestwick, Anthony J Richardson, Nest McKain, Tony R Larson, Ian A Graham, Robert J Wallace

**Affiliations:** 1 Rowett Institute of Nutrition and Health, University of Aberdeen, Bucksburn, Aberdeen AB21 9SB, UK; 2 Unidade de Produção Animal, Instituto Nacional de Recursos Biológicos, Fonte Boa, 2005-048 Vale de Santarém, Portugal; 3 Department of Biology, University of York, PO Box 373, York YO10 5YW, UK; 4 Current address: Requimte, ICBAS, Instituto de Ciências Biomédicas Abel Salazar, Universidade do Porto, Campus Agrário de Vairão, Rua Padre Armando Quintas, 4485-661 Vairão VC, Portugal; 5 Current address: Centre of Advanced Studies in Animal Nutrition, Indian Veterinary Research Institute, Izatnagar - 243 122, India

## Abstract

**Background**

Health-promoting polyunsaturated fatty acids (PUFA) are abundant in forages grazed by ruminants and in vegetable and fish oils used as dietary supplements, but only a small proportion of PUFA finds its way into meat and milk, because of biohydrogenation in the rumen. *Butyrivibrio fibrisolvens *plays a major role in this activity. The aim of this study was to investigate the mechanisms by which PUFA affect the growth of *B. fibrisolvens*, how PUFA are metabolized and the metabolic response to growth in the presence of PUFA.

**Results**

Linoleic acid (LA; *cis*-9, *cis*-12-18:2) and α-linolenic acid (LNA; *cis*-9, *cis*-12, *cis*-15-18:3) increased the lag phase of *B. fibrisolvens *JW11, LNA having the greater effect. Growth was initiated only when the PUFA had been converted to vaccenic acid (VA; *trans-*11-18:1). The major fish oil fatty acids, eicosapentaenoic acid (EPA; 20:5(*n-*3)) and docosahexaenoic acid (DHA; 22:6(*n-*3)), were not metabolized and prevented growth. Cellular integrity, as determined fluorimetrically by propidium iodide (PI) ingression, was affected as much by 18:1 fatty acids, including VA, as 18:2 fatty acids. The methyl esters of LNA, LA, EPA and DHA had no effect on growth or other measurements. The ATP pool decreased by 2/3 when LA was added to growing bacteria, whereas most acyl CoA pools decreased by >96%.

**Conclusions**

It was concluded that biohydrogenation occurs to enable *B. fibrisolvens *to survive the bacteriostatic effects of PUFA, and that the toxicity of PUFA is probably mediated via a metabolic effect rather than disruption of membrane integrity.

## Background

Unsaturated fatty acids, particularly α-linolenic acid (LNA; *cis*-9, *cis*-12, *cis*-15-18:3) and linoleic acid (LA; *cis*-9, *cis*-12-18:2), are abundant in grass and other ruminant feedstuffs, yet are present at low concentrations in meat and milk. Furthermore, tissue lipids of ruminants have been known for a long time to be more saturated than those of non-ruminants [1]. As the consumption of saturated acids in dairy products and ruminant meats is often associated with an increased incidence of coronary heart disease in man [2], the transformation of unsaturated fatty acids to saturated fatty acids, or biohydrogenation, in ruminants presents a major human health issue. The biohydrogenation process has long been known to occur in the rumen as the result of microbial metabolic activity [3,4]. Thus, if ruminal biohydrogenation of unsaturated fatty acids can be controlled, it may be possible to improve the healthiness of ruminant meats and milk by increasing their unsaturated fatty acids composition in general and the *n*-3 fatty acids in particular [5]. One of the unsaturated fatty acids that appears most desirable is conjugated linoleic acid (CLA; *cis*-9, *trans*-11-18:2) because of its anticarcinogenic and other health-promoting properties [6,7]. Major advances have been made in achieving the desired changes in fatty acid content of meat and milk experimentally, via dietary manipulation in ruminants, generally by adding oils containing unsaturated fatty acids to the diet [5,8-10]. The inclusion of fish oil in particular seems to alter biohydrogenating activity in the rumen [11].

*Butyrivibrio fibrisolvens *was identified many years ago to undertake biohydrogenation of fatty acids [12] and to form CLA as intermediate in the process [13]. Kim *et al*. [14] noted that LA inhibited growth of *B. fibrisolvens *A38, an effect that depended both on the concentration of LA and the growth status of the bacteria. Growing bacteria were more tolerant of LA. In a study of CLA production in different strains of *B. fibrisolvens*, Fukuda *et al*. [15] found that the most tolerant strain had the highest linoleate isomerase (forming CLA from LA) specific activity. Different members of the *Butyrivibrio*/*Pseudobutyrivibrio *phylogenetic grouping, all of which biohydrogenate PUFA, had different sensitivities to growth inhibition by LA, the most sensitive possessing the butyrate kinase rather than the acyl transferase mechanism of butyrate production [16]. For reasons that were unclear, lactate exacerbated the toxicity of LA to *Clostridium proteoclasticum *[17], now renamed *Butyrivibrio proteoclasticus *[18]. The aim of this work was to understand the nature of the toxic effects of PUFA on *B. fibrisolvens *JW11. Strain JW11 is located in the middle of the numerous *B. fibrisolvens*/*Pseudobutyrivibrio *cluster, members of which share the ability to form CLA and vaccenic acid (VA; *trans*-11-18:1) but which also lack the ability to biohydrogenate VA to stearic acid (SA; 18:0) [16]. Understanding these effects could have important indirect implications for human health by enabling ruminal biohydrogenation of dietary PUFA to be manipulated in order to provide healthier ruminant-derived foods.

## Results

### Fatty acid metabolism by *B. fibrisolvens *JW11

**Figure 1:**
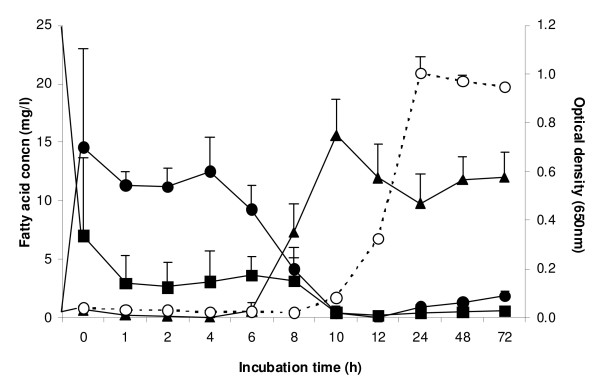
**Concentration of fatty acids in the medium following inoculation of *B. fibrisolvens *JW11 into M2 medium containing 50 μg ml^-1 ^linoleic acid (LA; *cis*-9, *cis*-12-18:2)**. Growth (open circle, OD_650_), LA (square), *cis*-9, *trans*-11-18:2 (black circle), *trans*-11-18:1 (triangle). Results are means and SD from three cultures.

The metabolism of LA was measured during the growth cycle of *B. fibrisolvens *JW11 (Figure 1). No growth occurred until 10 h, but then growth was initiated and bacteria grew at a specific growth rate similar to that found in the absence of added fatty acid (not shown). During the lag phase, LA was very rapidly converted to CLA, but growth was not initiated until all the dienoic acids had been metabolized and converted extensively to vaccenic acid. No SA was formed.

**Figure 2:**
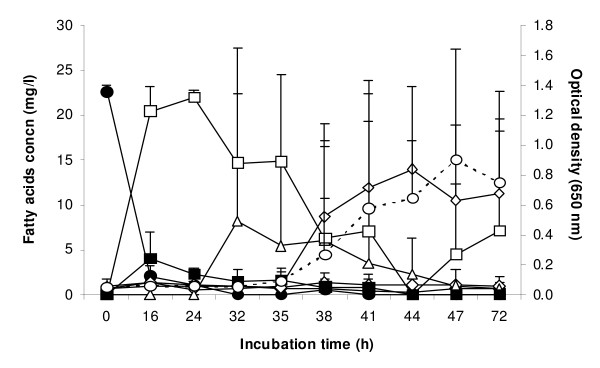
**Concentration of fatty acids in the medium following inoculation of *B. fibrisolvens *JW11 into M2 medium containing 50 μg ml^-1 ^α-linolenic acid (LNA; *cis*-9, *cis*-12, *cis*-15-18:3)**. Growth (open circle, OD_650_), LNA (black circle), *cis-*9, *trans-*11, *cis*-15-18:3 (open square), *trans*-9, *trans*-11, *cis*-15-18:3 (black square), *trans*-11, *cis*-15-18:2 (open triangle), *cis*-9, *trans*-11-18:2 (black triangle), *trans*-11-18:1 (diamond). Results are means and SD from three cultures.

A longer lag phase was seen with LNA (Figure 2). LNA was also metabolised rapidly during early lag phase, being converted firstly to the conjugated *cis*-9, *trans*-11-*cis*-15-18:3. A little *trans*-9, *trans*-11, *cis*-15-18:3 was formed as well. The main dienoic acid formed transiently was *trans*-11, *cis*-15-18:2, which was subsequently converted to VA. Variation in the time taken for different replicate tubes to escape the lag phase meant that the average concentration across three tubes gives a misleading impression. For example, at 32 h, replicate tubes contained 0.125, 0.140 and 0.193 mg bacterial protein ml^-1^, indicating that the culture in the third tube had begun to grow sooner than the others. The concentrations of *cis-*9, *trans*-11, *cis*-15-18:3 were 23.0, 21.1 and 0 μg ml^-1^, respectively, while the concentrations of *trans*-11, *cis*-15-18:2 were 0, 0 and 24.5 μg ml^-1^. An analysis comparing bacterial protein concentrations and fatty acid concentrations in the same tubes (not shown) demonstrated that bacterial protein concentration was low while *cis-*9, *trans*-11, *cis*-15-18:3 and *trans-*9, *trans*-11, *cis*-15-18:3 were present. Higher bacterial concentrations occurred only when these fatty acids were removed from individual cultures. High concentrations of VA did not affect growth, while *trans*-11, *cis*-15-18:2 also appeared to permit growth. No SA was formed in any LNA-containing culture.

### Comparative effects of fatty acids and methyl esters on growth and metabolism by *B. fibrisolvens *JW11

The effects of various fatty acids and their methyl esters on the growth of *B. fibrisolvens *and the biohydrogenation products in M2 medium were carried out in a similar way, and are summarized in Table 1. The more unsaturated fatty acids were more toxic, with γ-linolenic acid (γ-LNA; *cis*-6, *cis*-9, *cis*-12-18:3) and the fish oil fatty acids, DHA and EPA, causing lag phases >72 h. LNA induced a lag phase of 37 h, longer than that found with LA (*P *= 0.001), which in turn was longer than that caused by CLA (*P *= 0.01). VA and SA had little growth-inhibitory activity, while oleic acid (OA; *cis*-9-18:1) caused a short lag of just under 2 h (*P *= 0.005). γ-LNA was metabolized to a trienoic acid, which from its elution time in GC was judged to be conjugated, most likely *cis*-6, *cis*-9, *trans*-11-18:3. OA was not metabolised and was slightly toxic. Neither EPA nor DHA was metabolised. SA did not cause a lag phase and was not metabolised. No methyl esters caused a lag phase, yet they were converted to the same products as the free fatty acids, with the exception of methyl-γ-LNA, which formed a dienoic acid eluting in the same area as CLA. GC analysis of extracted samples before they were methylated indicated that the fatty acid methyl esters had been hydrolysed before the fatty acids were metabolized.

**Table 1:** Effects of fatty acids and methyl esters (50 μg ml^-1^) on growth of, and metabolism of fatty acids by, *Butyrivibrio fibrisolvens *JW11 in M2 medium.

	DHA	EPA	γ-LNA	LNA	LA	CLA	VA	OA	SA
**Free fatty acids**									
Lag phase (h)									
Mean	>72	>72	>72	37.0	7.1	4.7	0.01	1.88	0.82
SD				5.25	0.60	0.20	0.13	0.23	0.48
Biohydrogenation	No	No	Yes	Yes	Yes	Yes	No	No	No
End product at 72 h	ND^a^	ND	Conjugated 18:3	VA	VA	VA	ND	ND	ND
									
**Methyl esters**									
Lag phase (h)									
Mean	NA	NA	NA	NA	0.37	-0.17	-0.23	-0.07	1.54
SD					1.12	0.44	0.35	0.09	0.23
Biohydrogenation	No	No	Yes	Yes	Yes	Yes	No	No	No
End product at 72 h	ND	ND	Conjugated 18:2	VA	VA	VA	ND	ND	ND

^a^ND - None detected

^b^NA - Not analyzed. The first OD reading was made at 16 h, by which time the cultures had grown.

### Influence of fatty acids on cell integrity of *B. fibrisolvens *JW11

**Figure 3:**
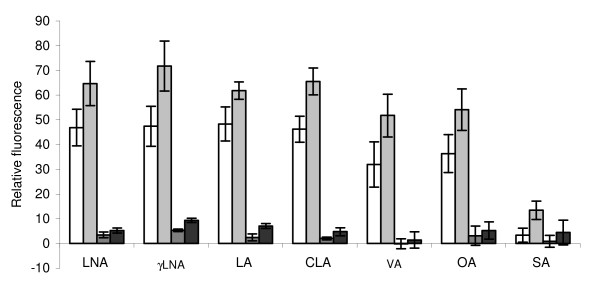
**Influence of different fatty acids and fatty acid methyl esters on cell integrity of *B. fibrisolvens *JW11**. Loss of cell integrity was determined fluorimetrically by propidium iodide fluorescence. LNA, *cis*-9, *cis*-12, *cis-*15-18:3; γLNA, *cis*-6, *cis*-9, *cis*-12-18:3; LA, *cis*-9, *cis*-12-18:2; CLA, a mixture of *cis*-9, *trans*-11-18:2 and *trans*-10, *cis*-12-18:2; VA, *trans*-11-18:1; OA, *cis*-9-18:1; SA, 18:0. In order of increasing shading density: 50 μg fatty acid ml^-1^, 200 μg fatty acid ml^-1^, 50 μg fatty acid methyl ester ml^-1^, 200 μg fatty acid methyl ester ml^-1^. Results are means and SD from three determinations.

The influence of different C-18 fatty acids and their methyl esters on cell integrity was determined using propidium iodide (PI) fluorescence (Figure 3). All unsaturated fatty acids, including OA and VA, had a similar effect, although the monoenoic acids tended to cause less disruption (*P = *0.063). The effects of 200 μg ml^-1 ^fatty acids were only slightly greater than 50 μg ml^-1 ^(*P *< 0.001). SA gave a small response in comparison with the unsaturated fatty acids (*P *< 0.001), and methyl esters caused only about one-tenth of the disruption of the free fatty acids (*P *< 0.001) (Figure 3).

**Figure 4:**
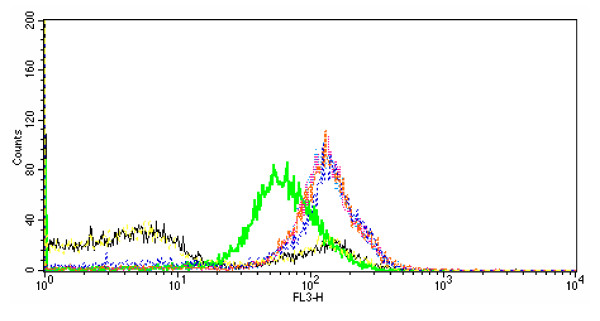
**Influence of different fatty acids on PI fluorescence of *B. fibrisolvens *JW11 by flow cytometry**. Black - live cells; green - heat-killed cells; pink - 50 μg ml^-1 ^LA; turquoise - 50 μg ml^-1 ^LNA; orange - 50 μg ml^-1 ^CLA; blue - 50 μg ml^-1 ^VA; yellow - 50 μg ml^-1 ^SA.

The influence of fatty acids on cell integrity was analysed further by flow cytometry (Figure 4). All unsaturated fatty acids transformed the PI signal to one in which the great majority of cells displayed fluorescence, i.e. the fluorescence response profile moved to the right in the flow display. The unsaturated fatty acids caused apparently greater disruption than boiling the cells, suggesting that the fatty acids enhanced access of PI to the bacterial cytoplasm. SA had no effect, the profile following exactly that of untreated cells. Differences between the different unsaturated fatty acids were minor. Even in untreated cell suspensions, some fluorescence was observed at the 10^2 ^region, consistent with about 25% of the bacteria being non-viable. Very few cells remained unaffected by either boiling or the fatty acids, judging by the low incidence of fluorescence at the <10^1 ^region of the traces.

**Figure 5:**
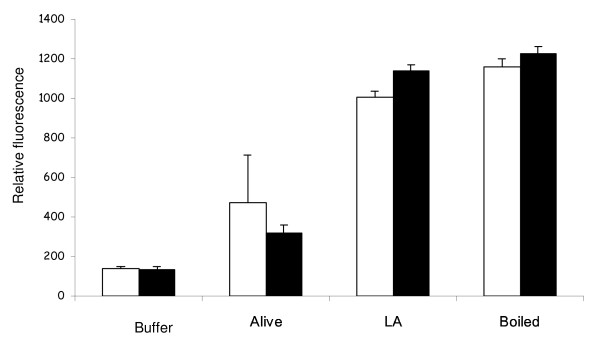
**Influence of sodium lactate (70 mM) on the loss of cell integrity of *B. fibrisolvens *JW11 following incubation with LA (50 μg ml^-1^)**. Loss of cell integrity was determined by fluorescence in the presence of propidium iodide. Sodium lactate + LA (open bar), LA alone (black bar). Results are means and SD from three cultures, each of which was subject to 8 replicate measurements (*n *= 24).

The presence of 70 mM sodium lactate in the growth medium increased the lag phase from 7 to 16 h (not shown) when LA was present. The influence of LA on PI fluorescence and growth was also determined in the presence and absence of sodium lactate (Figure 5). As before, LA increased the fluorescence due to PI (*P *< 0.001), indicating that cell integrity had been disrupted. Sodium lactate did not alter the response significantly (*P *> 0.05).

### Influence of LA on ATP and acyl CoA pools of *B. fibrisolvens *JW11

**Figure 6:**
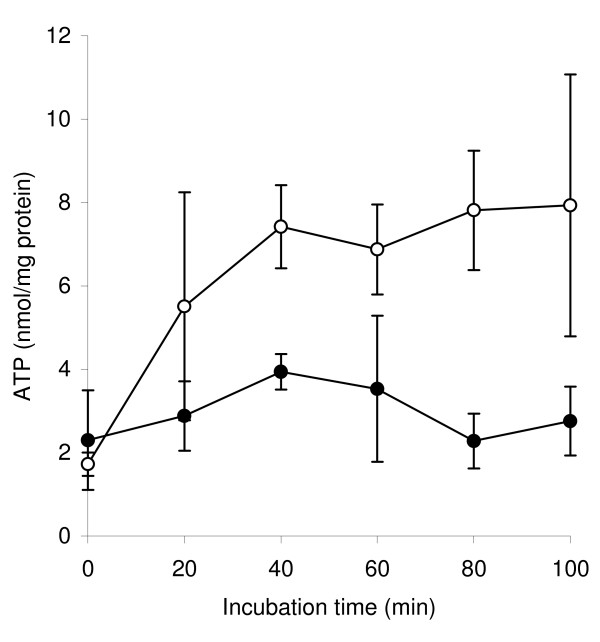
**Influence of LA on ATP pools of *B. fibrisolvens *JW11 after 50% inoculation into fresh medium**. LA (black circle), no LA (open circle). Results are means and SD from three separate cultures.

LA was added to culture that had been mixed with an equal volume of fresh medium, and the pool sizes of ATP (Figure 6) and acyl CoA intermediates (Table 2) were measured. Initially, the ATP pools were similar, at about 2 nmol (mg protein)^-1^. Thereafter, the ATP pool remained similar in the LA culture, while the concentration increased 3-4-fold (*P *< 0.05 from 40 min onwards) in cultures to which no LA was added. The acyl CoA pools were measured only after 20 min, at which time the ATP pool had not yet changed significantly (*P *> 0.05). In control cultures, the highest pool sizes of short-chain acyl CoAs were of acetyl CoA and butyryl CoA, followed by propionyl CoA. Crotonyl CoA and acetoacetyl CoA were present at much lower concentrations, 10 pmol (mg protein)^-1 ^or less. β-Hydroxybutyryl CoA was not determined by the methods used. All CoA pools, except acetoacetyl CoA, were decreased by >96% (*P *< 0.001) in LA-containing cultures.

**Table 2:** Influence of LA on acyl CoA pools of *B. fibrisolvens *JW11 20 min after inoculation into fresh medium.

	Acyl CoA concentration (pmol mg protein^-1^)			
	
Acyl CoA	No addition		0.2 mg ml^-1^LA	
		
	Mean	SD	Mean	SD
Acetyl	375	158	17	5
Propionyl	53	14	2	1
Isobutyryl	16	4	0	0
Butyryl	213	77	10	2
Crotonyl	10	6	0	0
Isovaleryl	8	2	0	0
Hexanoyl	2	1	0	0
Acetoacetyl	4	1	7	1

Results are means and SD from three separate cultures.

## Discussion

*B. fibrisolvens *was originally described as a small, Gram-positive bacterium particularly prevalent in the rumen of grazing animals [19]. Many strains are proteolytic and involved in fibre breakdown [19,20]. *B. fibrisolvens *JW11 was originally isolated as a proteolytic strain [21]. It has been many years since the importance of *B. fibrisolvens *in the process of PUFA reduction, or biohydrogenation, was first documented [12]. Although other bacteria have been implicated [22], biohydrogenating activity is high among all members of what is now known to be an extensive *Butyrivibrio *phylogenetic tree [16]. Indeed, in our experience, its activity is many times higher than in other species [17]. 'Type B' bacteria, which complete the reduction of 18:1 isomers to SA, was identified as *C. proteoclasticum *[23], which has recently been renamed *Butyrivibrio proteoclasticus *[18]. The pattern of metabolism of LA and LNA observed here, and the identity of the intermediates, follows the pathways established first by Kepler *et al*. [13] and confirmed later by others [24-26]. The observations linking growth and LA metabolism with *B. fibrisolvens *JW11 are consistent with those obtained with *B. fibrisolvens *A38 [14] and *B. fibrisolvens *TH1 [15]. What is novel about the present observations is that they clearly demonstrate that biohydrogenation is a detoxification process, necessary to escape from the bacteriostatic effects of PUFA. Indeed, they explain that the concentration-dependence of LA toxicity and its apparently lower toxicity in growing cultures, observed by Kim *et al*. [14], have to be considered in terms of time required by different biomass concentrations to hydrogenate, and thereby detoxify, different concentrations of fatty acids.

Henderson [27] examined the effects of fatty acids on ruminal bacteria. A *Butyrivibrio *sp. was generally most sensitive to fatty acids, but only saturated and monoenoic acids were included in the study. OA was much more toxic than the saturated fatty acids. Marounek *et al*. [28] found that C-12 and C-14 fatty acids were more toxic to ruminal and rabbit caecal bacteria than other chain lengths, but again the study was of saturated acids and oleic acid. In non-ruminal bacteria, LA and LNA were much more toxic than saturated or monoenoic acids [29]. The present paper describes the effects of the more abundant poly- and monounsaturated fatty acids on *B. fibrisolvens*. The PUFA were found to be much more toxic than more saturated fatty acids.

The present experiments help to resolve the purpose of biohydrogenation in the ruminal bacteria that undertake this reductive metabolism. Our results provide support for the conclusions of Harfoot and Hazlewood[22], Kemp and Lander [30] and Kemp *et al*. [31] that biohydrogenation is a detoxification mechanism rather than a means of disposing of reducing power, as proposed earlier [32]. The reductase which converts CLA to VA in *B. fibrisolvens *comprises 0.5% of the total cell protein [33], a very significant expenditure of cellular resources that signifies a vital function. It should be noted that, although more research emphasis is placed on its metabolism of LA because CLA is an intermediate, biohydrogenation is probably more important for *B. fibrisolvens *to survive high LNA concentrations, as LNA is more toxic than LA and is usually present at higher concentrations than LA in forages (e.g. [3]). Also to be noted is that CLA is almost as toxic as LA, as found before [14].

There are several possible reasons why unsaturated fatty acids are generally more toxic than saturated fatty acids. The double bonds alter the shape of the molecule, such that kinked unsaturated fatty acids disrupt the lipid bilayer structure [34]. The finding that different PUFA isomers, such as LNA and γ-LNA, had different toxicity would be consistent with such an interpretation. However, it is not clear that the toxicity was necessarily a membrane effect. The free carboxyl group was necessary for growth inhibition to take place. Methyl esters, which might be expected to be sufficiently hydrophobic to be incorporated into a membrane just as efficiently as a free fatty acid, were non-toxic. They were metabolized in the same way as the free fatty acids, however, as they were hydrolysed by bacterial esterase activity. The free carboxyl group was also necessary for disruption of cell integrity, as measured by PI ingression. However, PI ingression was affected little by the number of double bonds in the fatty acid molecule, as detected by both fluorescence spectroscopy and flow cytometry, and could not explain why 18:3 fatty acids were more toxic to growth than 18:2 fatty acids, and especially why 18:1 was not toxic to growth. Furthermore, sodium lactate exacerbated growth inhibition by LA, in a similar manner to that observed with *B. proteoclasticus *[23], but had no similar effect on the influence of LA on cell integrity of *B. fibrisolvens*. A similar conclusion was reached by Maia *et al*. [17] when comparing the toxic effects of fatty acids on growth and cell integrity in different species of ruminal bacteria. Thus, although a toxic mechanism involving disruption of the extraordinarily thin cell envelope of *B. fibrisolvens *[35] seems an attractive and logical possibility, the evidence suggests that the primary effect of PUFA lies elsewhere.

An alternative possibility is that the ready diffusion of the free fatty acid across the membrane causes chemiosmotic difficulties, perhaps uncoupling the proton-motive force [36], dissipating the membrane potential by facilitating ion leakage [37] or decoupling intramembrane pathways [38,39]. While this remains a possibility, the different effects on acyl CoA and ATP pools on PUFA toxicity suggest a metabolic effect, specifically in acyl CoA metabolism. Measurement of CoA metabolic pools in bacteria is relatively rare. Here, acetyl CoA and butyryl CoA were present at highest concentration and the butyrate pathway intermediates at much lower concentrations, as found also in *Clostridium acetobutylicum *[40]. All acyl CoAs except acetoacetyl CoA were diminished by >96% when LA was added to the medium. In contrast, the ATP pool was affected later than acyl CoA pools, and remained at about one-third of the control values, presumably due to the contribution of glycolysis.

The toxicity of PUFA in different species of ruminal bacteria was found to be related partly to whether the bacteria produced butyrate; cellulolytic bacteria were the other most sensitive species [17]. Within the *Butyrivibrio *phylogenetic group, the most sensitive species were those that formed butyrate *via *the butyrate kinase mechanism rather than acyl CoA transferase [16]. Thus, there seems to be a connection between PUFA toxicity and butyrate formation. A metabonomic analysis [41] might help to identify precisely where the PUFA act. It may also be instructive to determine why *trans*-11, *cis*-15-18:2, a product of LNA metabolism, appeared to permit growth while the other dienoic acid investigated here did not.

The influence of sodium lactate in lengthening the lag phase indicates that lactate potentiates the toxic effects of PUFA in *B. fibrisolvens*, as shown previously with *B. proteoclasticus *[22]. Such a high concentration of lactate (70 mM) would only occur in animals suffering acidosis [42]. The toxicity may be an osmotic effect, or due to a leakage of ions across the membrane, or may even be a metabolic effect. Lactate is a major product of glucose metabolism in *B. fibrisolvens *JW11 and many other butyrivibrios [16], and high concentrations may therefore impose metabolic feedback, further stressing bacteria affected by PUFA.

## Conclusions

This paper explains the basis of the beneficial effect on meat and milk fatty acid composition of adding oils to the ruminant diet. Ruminal biohydrogenation is modified via differential toxicity to ruminal bacteria of different PUFA, including the fish oil fatty acids, EPA and DHA. If we can understand how selective fatty acid toxicity, or indeed other factors, affects the physiology of biohydrogenating bacteria in the rumen, we may be able to suggest new, rational dietary modifications that will eventually lead to ruminant products that are healthier for human consumption.

## Methods

### Bacteria and growth conditions

*Butyrivibrio fibrisolvens *JW11 was originally isolated from sheep as a proteolytic species [21], and is held in the culture collection maintained at the Rowett Institute. All transfers and incubations were carried out under O_2_-free CO_2 _and at 39°C in Hungate-type tubes [43]. Inoculum volumes were 5% (v/v) of a fresh culture. The media used in these experiments were the liquid form of M2 medium [44]. Fatty acids were prepared as a separate solution, sonicated for 4 min in water and added to the medium before autoclaving. Growth of bacteria was measured from the increase in optical density (OD) at 650 nm of the control tubes, in triplicate, using a Novaspec II spectrophotometer (Amersham Biosciences, UK).

The influence of fatty acids and their methyl esters was determined in two kinds of experiment. In experiments where fatty acid concentrations were measured at the end-point of the growth curve, usually in stationary phase, the tubes were freeze-dried in order to enable fatty acid extraction from the whole culture. The experiment was conducted by inoculating multiple 10-ml tubes. At each sampling time, three tubes were removed, the turbidity was determined, and the tubes were placed in a heating block at 100°C for 5 min, left to cool and frozen. One ml was taken for protein analysis and for fatty acid extraction and derivatization.

### Fatty acid extraction and analysis

Extraction, derivatization of fatty acids and GC analysis of methyl esters were carried out using procedures described by Wąsowska *et al*. [11]. The products from incubations with LNA were identified by comparing elution profiles and mass spectra with those identified previously from analysis of methyl and 4,4-dimethyloxazoline (DMOX) esters [11].

### Measurement of cell integrity using propidium iodide

One ml of overnight culture was inoculated into 10 ml of M2 medium and incubated at 39°C until it reached mid-exponential phase (OD_650 _= 0.4, approx. 4 h). The bacterial cultures were centrifuged (3000 ***g***, 10 min, 4°C) and the pellet was washed twice with anaerobic potassium phosphate buffer (100 mM; pH 7.0) containing 1 mM dithiothreitol (DTT). Anaerobic conditions were maintained by carrying out transfers in an anaerobic chamber with a gas phase of 80% N_2_, 10% CO_2 _and 10% H_2 _and temperature of 39°C. Cells were resuspended in 15 ml of the same buffer, and fatty acids and their respective methyl esters (Sigma, St. Louis, MO, USA) were added to the cell suspension to a final concentration of 50 μg ml^-1^. Stock solutions (1 mg ml^-1^) of fatty acids and methyl esters were prepared immediately before use by sonication for 4 min in anaerobic potassium phosphate buffer (100 mM, pH 7.0, containing 1 mM DTT). Untreated and heat-treated cells (100°C for 20 min) served as control samples.

Following 30 min incubation of cell suspensions with fatty acids, cell integrity was measured using PI. Ten μl of each sample were added to 985 μl of anaerobic potassium phosphate buffer, to which was added 5 μl of 1.5 mM PI (prepared in distilled water and stored at 4°C in the dark). The mixtures were incubated for 15 min at 39°C in the anaerobic chamber, then transferred to an ice-water slurry and kept in the dark for up to 45 min before being analysed for fluorescence using a fluorimeter or by flow cytometry. Fluorimetry measurements were made using a spectrofluorimeter set at λ_EX _= 488 nm and λ_EM _= 650 nm. Flow cytometry was carried out with a FACSCalibur flow cytometer (Becton Dickinson Immunocytometry Systems, San Jose, California, USA) equipped with an air-cooled argon ion laser emitting 15 mW of blue light at 488 nm. The red fluorescence of the PI signal was collected in the FL3 channel (>600 nm long-pass filter). FACSFlow solution (Becton Dickinson) was used as sheath fluid. The analyses were done using the low rate settings (12 μl/min).

### ATP and acyl CoA pools

The influence of LA on metabolic pools in *B. fibrisolvens *was measured in cells growing in Roché *et al*. [45] medium in the anaerobic chamber, as follows. Fresh overnight culture (60 ml) of *B. fibrisolvens *JW11 was mixed with 60 ml of uninoculated medium, or uninoculated medium containing 200 μg LA ml^-1^, then samples (3.0 ml) were taken periodically into 1 ml of 30% (w/v) perchloric acid. After 10 min, 4 ml of KOH were added to the acidic solution, forming a precipitate of potassium perchlorate, which was removed by centrifugation (15,000 ***g***, 15 min, 4°C). The supernatant was stored at -80°C, then subsequently thawed and ATP was measured using a luciferase preparation according to the manufacturer's (Sigma) instructions.

Acyl CoA measurements were made in parallel 120-ml control or LA-containing cultures after 20 min incubation. Cultures were maintained under CO_2 _and centrifuged immediately at 15,000 ***g ***for 15 min at 39°C. The pellet was stored in liquid nitrogen. Derivatization, separation, and fluorescence detection of acyl CoAs were carried out as described by Larson and Graham [46]. Identification of acyl CoAs was carried out using mass spectrometric analysis of peaks obtained from a Hypercarb porous graphitic carbon column [47].

Bacterial protein was measured by a modification of the Lowry method [48].

### Data analysis

All data were derived from three separate cultures. Means were compared by a Student's *t*-test. Measurement of the lag phase was carried out by fitting a gradient by linear regression to log(*A*_650_) *vs*. time during exponential phase. The lag phase was defined as the time at which the best-fit gradient passed an OD_650 _of 0.1, and was compared to the time at which the control cultures passed 0.1. Propidium iodide ingression was determined by 8 fluorescence measurements for each culture.

## List of abbreviations

CLA: conjugated linoleic acid; DHA: docosahexaenoic acid; DMOX: 4,4-dimethyloxazoline; DTT: dithiothreitol; EPA: eicosapentaenoic acid; LA: linoleic acid; LNA: α-linolenic acid; γ-LNA: γ-linolenic acid; OA: oleic acid; OD: optical density; PI: propidium iodide; PUFA: polyunsaturated fatty acids; SA: stearic acid; VA: vaccenic acid.

## Authors' contributions

MRGM designed and carried out cell integrity studies, some growth experiments, and assisted in drafting the manuscript. LCC carried out growth experiments and fatty acids analysis. CSB participated in the design and implementation of flow cytometry experiments and in discussion of bacterial viability. AJR carried out experiments on metabolic pools, and assisted in drafting the manuscript. NM supervised growth experiments, fatty acids analysis and assisted in drafting the manuscript. TRL and IAG undertook the analysis of acyl CoAs. RJW designed the studies, collated the experimental data and wrote the manuscript.

All authors read and approved the final manuscript.
